# The influence of contact lenses with different optical designs on the binocular vision and visual behavior of young adults

**DOI:** 10.1038/s41598-022-10580-7

**Published:** 2022-04-21

**Authors:** Shyan-Tarng Chen, Hsiao-Ching Tung, Yan-Ting Chen, Chuen-Lin Tien, Chih-Wei Yeh, Jheng-Sin Lian, Ching-Ying Cheng

**Affiliations:** 1 Department of Optometry, Chung Shan Medical University, Taichung, Taiwan; 2 Department of Ophthalmology, Chung Shan Medical University Hospital, Taichung, Taiwan; 3 Dr. Don’s Eye Clinic, Taipei, Taiwan; 4 Department of Electrical Engineering, Feng Chia University, Taichung, Taiwan; 5 Shu-Zen Junior College of Medicine and Management, Kaohsiung, Taiwan

**Keywords:** Health care, Health occupations

## Abstract

Theoretically, contact lenses change the accommodation and vergence demands of the eyes and directly or indirectly influence binocular vision. The purpose of this study was to investigate the influence of different optical designs of contact lenses on binocular vision and visual behavior among young adults. In this randomized, single-blinded experimental study, visual behavioral performance (VBP) and Ocular Surface Disease Index questionnaires were used for preliminary screening. Nineteen myopic subjects aged 20–26 years (21.59 ± 1.40 years) participated in the study. Baseline values for subjective refraction and binocular visual functions were evaluated. All the subjects were randomly grouped to wear different optical designs of single-vision, progressive, and bifocal contact lenses. Eye examinations were performed on the first day (CL baseline), 2nd week, and 4th week of wearing the lenses. In addition, subjects completed the VBP and visual quality questionnaire again at the end of the examination. Single-vision contact lenses (Lens 1) increased accommodative lag, negative relative accommodation, and distant and near vergence; however, vergence facilities, accommodative facilities, heterophoria, and the comfort and posture balance dimensions in the VBP questionnaire were improved. Progressive contact lenses (Lens 2) reduced the lag of accommodation and near vergence; in addition, vergence facilities and accommodative facilities were also improved. Bifocal contact lenses (Lens 3) affected negative and positive relative accommodation, but vergence facilities and accommodative facilities also progressed. Furthermore, both progressive (Lens 2) and bifocal (Lens 3) contact lenses enhanced overall visual behavioral performance. In terms of visual quality, single-vision contact lenses (Lens 1) were the most comfortable, progressive and bifocal contact lenses reduced distant visual acuity and stability, progressive contact lenses (Lens 2) had more complaints about halos at night, and bifocal contact lens (Lens 3) users were more likely to have double vision. Compared with single-vision contact lenses, progressive and bifocal contact lenses relaxed accommodation, reduced the lag of accommodation, and improved visual behavioral performance. Although the vergence function showed a significant change, it did not show worse trends when wearing contact lenses. Contact lenses with different optical designs have a great influence on binocular vision and visual behavioral performance.

## Introduction

With technological advances and changes in lifestyle, the global myopia rate has rapidly increased annually. It is estimated that 50% of the global population will be myopic by 2050^1^. The correlation between contact lenses and myopia progression has also gradually attracted attention. Contact lenses with different optical designs have been successively launched, of which orthokeratology, concentric bifocal soft contact lenses, and multifocal soft contact lenses have demonstrated some degree of effectiveness in controlling myopia in children^2–5^. Peripheral defocus in contact lenses is proved to influence the binocular vision significantly, researchers are planning to design contact lenses with gradient strengths of peripheral defocus to test and quantify the strength required to remedy or improve certain binocular dysfunctions to facilitate visual training or better control of myopia progression. However, the mechanisms by which these lenses control myopia progression are still unknown, and the main hypotheses of the present study were the concepts of accommodation^6^ and retinal peripheral defocus.

In binocular vision, both eyes simultaneously receive equal stimuli and operate in coordination so that similar images with slight differences are formed in the retinas of each eye. Fusion of the images from both eyes and integration in the brain results in a single and clear image^7–9^. Binocular fusion occurs in a small portion of the visual space around the region where the eyes are fixating. Running through the fixation point in the horizontal plane is a curved line called the empirical horizontal horopter or Panum’s fusion area^9^. Traditional visual acuity and binocular fusion are defined for the central foveal retina, which has the best correctable vision for stereopsis. However, the central visual field is only a small fraction of the total visual field. Although peripheral visual field has a much lower resolution and correctable vision, the considerably wider field for fusion may considerably contribute to the maintenance of binocular fusion and visual efficiency^10,11^.

In addition to peripheral refraction and fusion, eye movement and vergence are used to maintain correspondence in both retinas. When fusion abnormalities occur, eye fatigue, headache, ophthalmodynia, blurred vision, and focusing difficulties^12,13^ may present. Severe cases may also demonstrate diplopia, strabismus, and suppression^7,9^. Visual efficiency is the eye's ability to track, converge, and focus quickly. Evaluation of visual efficiency is composed of four systems, including oculomotor, accommodative, vergence, and sensory systems. Visual efficiency is essential for suitably processing visual information and difficulty related to the same is generally referred to as a visual disturbance, which may additional problems in visual behavior performance^12,13^.

Early studies found that stimulation of the peripheral retina causes an accommodative response, which decreases with increase in retinal eccentricity. The level of accommodation may depend on peripheral contrast sensitivity and is affected by vergence in both eyes^14,15^. Peripheral contrast sensitivity is affected by optical defocus, and correction of peripheral refractive errors can increase contrast sensitivity^16^. Although it is still impossible to accurately measure the refractive errors of the peripheral retina, peripheral hyperopic defocus is often found in myopic eyes^17^. This finding is related to the concept of using specially designed lenses to correct peripheral refractive errors and to control myopia^15,18,19^.

Compared to eye frames, wearing single-vision contact lenses would increase accommodation and vergence demands when looking at objects placed in close proximity^20–22^, concurrently leading to changes in binocular vision^23^. The near-vision addition of multifocal contact lenses can assist in relaxation of the accommodative response and the tendency of exophoria^24^ in myopic children. When myopia control is not the main consideration, the results of previous studies highlighted that the use of multifocal contact lenses result in accommodation changes and exophoria shifts^24–27^, which may be beneficial to patients with accommodative insufficiency and vergence abnormalities^28^. In addition, multifocal contact lenses are used to correct peripheral refractive errors and result in uniform defocus close to the retina or even peripheral myopic defocus, thereby improving accommodative response and vergence in the eyes^24^.

**Figure 1:**
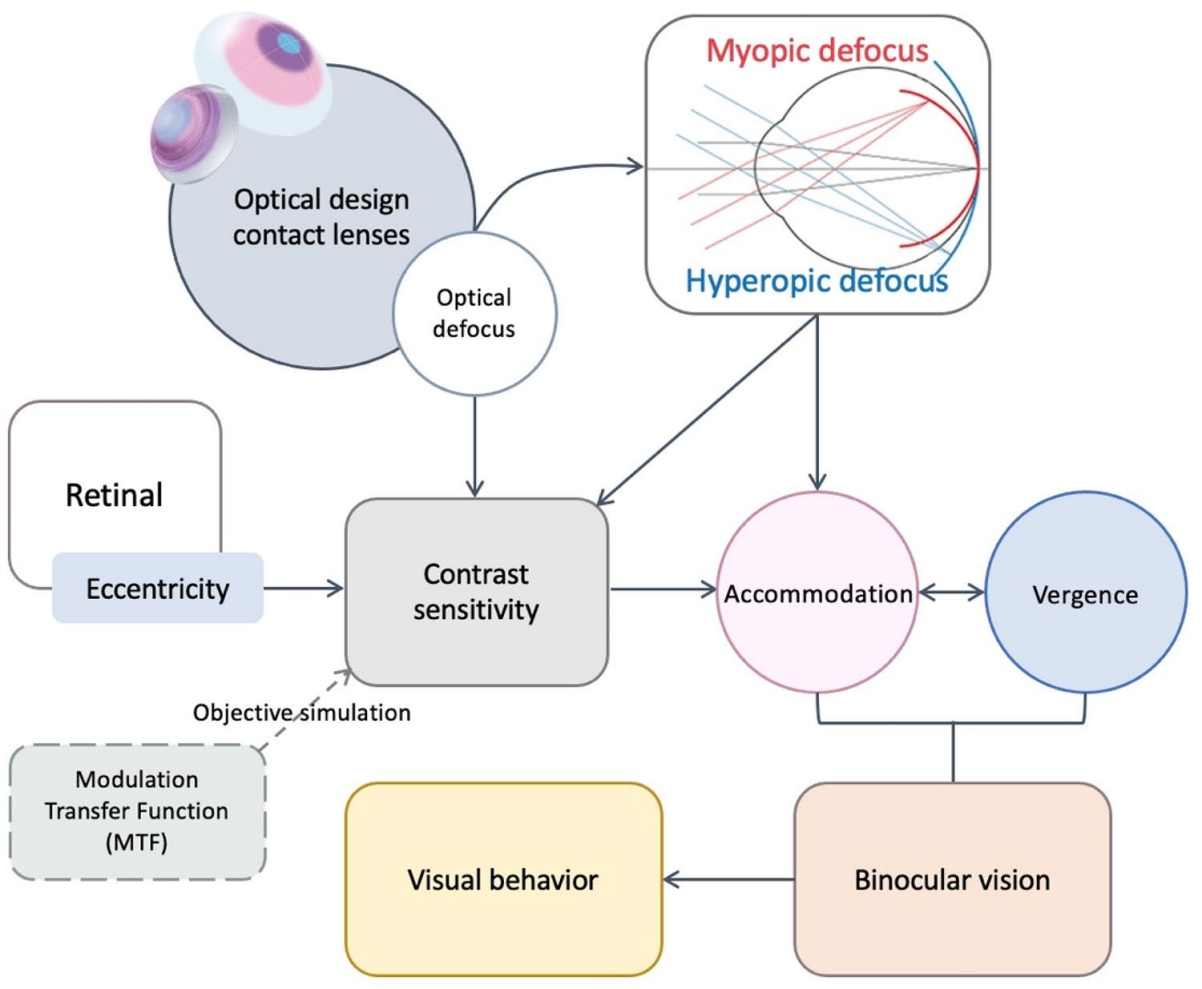
Relationship of Research hypothesis.

As shown in Fig. 1, the use of the modulation transfers function (MTF) can be based on wave front aberration to objectively simulate contrast sensitivity^29^. The literature indicates that peripheral retinal stimulation causes an accommodative response, peripheral contrast sensitivity changes with retinal eccentricity, and further affects the magnitude of the accommodative response^30,31^. The application of peripheral optical defocus in a contact lens for correction of hyperopia and myopia can change accommodative responses during defocus changes. The accommodative response is closely related to vergence, and both are important parameters in the evaluation of binocular vision for visual efficiency. The purpose of this study was to examine the influence of wearing contact lenses with different optical designs on binocular visual efficiency in adolescents.

## Materials and methods

### Study design

An experimental design study was performed from 18 November 2020 to 30 May 2021 at Chung Shan Medical University, Taichung, Taiwan. This experimental study protocol was reviewed and approved by the institutional review board of Chung Shan Medical University Hospital (approval no.: CS2-20089), and the study strictly adhered to the research ethics specifications in the Declaration of Helsinki. To ensure accurate measurement results, possible confounding factors such as laboratory brightness, visual target distance, and subjective differences in measurement tools and equipment operators were controlled. (STROBE guideline for reporting the manuscript^32,33^).

After the investigators had completely described the study content and procedure, subjects voluntarily decided to participate in the study and signed the informed consent form. The visual behavioral performance (VBP)^34,35^ and Ocular Surface Disease Index (OSDI) questionnaire were used for preliminary screening, and VBP and OSDI scores of ≥ 9 and < 13 points were included, respectively. Subjects who met the inclusion criteria showed no symptoms of dry eye but presented with abnormalities of the binocular visual to some extent.

### Participants

Taiwanese young adults aged 20–26 years were enrolled in this study. The study subjects were mainly from Chung Shan Medical University. The inclusion criteria in the study were refractive errors of myopia ≤ 6.00 D, astigmatism ≤ 1.00 D. Subjects with strabismus, eye disease, autoimmune disease, and who underwent eye surgery were excluded. Of the 24 people included after the initial screening, 5 were subsequently excluded—2 subjects were unable to adapt to wearing the contact lenses, 1 subject was unable to cooperate with the schedule, 1 subject had strabismus, and 1 subject had an astigmatism diopter that did not meet the criteria. Finally, 19 subjects with myopia with a mean age of 21.59 ± 1.40 years participated in this study.

Notably, subjects screened by VBP expressed visual disturbance, and 19 subjects in this study were classified according to Scheiman & Wick's criteria^36^. The results showed that there were two accommodative insufficient, one convergence excess, two convergences insufficient, and three exophoria. Although some subjects in this study could not be clearly classified into specific types of binocular visual abnormalities, their binocular visual functions continued to be different from the standard value to a certain extent. For patients with different types of binocular vision abnormalities, ideal optical design contact lenses that would be suitable will be discussed in the future.

### Materials

Three types of soft contact lenses were used as follows: Lens 1 (Lens Aqua Biweekly Soft Contact Lens [iLens]), Lens 2 (ArtMost Aspherical Soft Contact Lens [ArtMost]), and Lens 3 (SEED 1dayPure UP Multistage Daily Disposable Soft Contact Lens [SEED 1dayPure UP]). Table 1 shows the contact lens design and specification parameters; iLens had a spherical single-vision design; ArtMost had a center–distant aspherical multifocal design with progressive addition specification; and SEED 1dayPure UP had a center–distant concentric bifocal design. Considering that it was difficult for non-presbyopic subjects to adapt to a higher addition (+ 1.50 D) of bifocal lens (Lens 3), the specification of lower addition (+ 0.75 D) was finally selected to avoid causing distraction or image jump which was not an issue with progressive addition lenses. Each subject was asked to wear contact lenses for at least 6 h a day and, at each visit, we confirmed whether the subject followed this instruction. All the parameters were measured under the best correction. To avoid any potential sources of bias, each test was performed by the same optometrist.

**Table 1:** Contact lens design and specification parameters.

Contact lens	Optical design	ADD (D)	Material	Water content (%)	Basic curve (mm)	Diameter (mm)
Lens1: iLens	Spherical single-vision	−	Ocufilcon D	55%	8.6	14.4
Lens2: ArtMost	Aspherical Progressive (Distance–center)	2.00~3.00 R = 2 mm 8–12 7 mm	Ocufilcon D	55%	9.0	14.4
Lens3: SEED	Bifocal concentric (Distance–center)	+ 0.75	2-HEMA	58%	8.8	14.2

The VBP questionnaire was used as a screening tool and as a basis for the subjective responses before and after wearing the contact lenses. The VBP (Cronbach's alpha = 0.851) contained 48 questions in 4 dimensions, each question might meet two dimensions concomitantly: (1) near work (25 questions, Cronbach's alpha = 0.837); (2) perceptual (20 questions, Cronbach's alpha = 0.768); (3) comfort (14 questions, Cronbach's alpha = 0.705); and (4) balance (12 questions, Cronbach's alpha = 0.775) with good reliability and validity and had a strong correlation and predictive potential for binocular visual functions [^34,35^, Appendix 1]. The purpose of using the OSDI questionnaire was to eliminate interference variables and avoid problems of binocular vision and visual quality caused by dry eye^37^.

In this study, a Nidek OPD Scan 3 wavefront aberrometer (Nidek Inc., Tokyo, Japan) was used to measure refractive errors, corneal curvature, diameter, MTF, and pupil size in bright and dark rooms. Of these, corneal curvature, diameter, and pupil size were important reference information for contact lens fitting, data of 5-mm pupil were collected for analysis, whereas MTF was calculated by transferring different optical spatial frequencies through an optical systems and it displayed the simulated contrast sensitivity based on wavefront aberration. The MTF values were recorded as area ratio (%), which is defined by curves between areas and the closer the ratio is to 100%, the closer the patient’s eye is to being normal. Past MTF studies mostly emphasized on comparing different designs of multifocal or bifocal intraocular lenses^38^. Whether changes in MTF based on optically designed contact lenses can reflect subjective contrast sensitivity remains unknown.

A Topcon VT-10 phoropter (Topcon, Tokyo, Japan), a View-M digital visual acuity chart (Quan Chin Industrial Co., Taiwan), and a TMV near point card (Brighten Optix Co., Taiwan) were used to measure subjective refraction and visual acuity. Regarding binocular visual function examination, Manual Phoropter was used for distance and near horizontal vergence range, distance fixation disparity, fused cross cylindrical (FCC), and negative relative accommodation/positive relative accommodation (NRA/PRA). The Saladin near point balance card was used to measure near fixation disparity. Howell cards and Maddox Rods were used to measure distance and near phoria. The Royal Air Force Rule (RAF, (Bernell Co., Mishawaka, IN) was used to measure near point convergence (NPC) and near point accommodation. The Butterfly Stereo Acuity Test (Bernell Co., Mishawaka, IN) was used to measure stereopsis. Next, a ± 2.00 D flipper was used to measure monocular accommodative facility (MAF) and binocular accommodative facility (BAF), and a 12△base-out (BO)/3△base-in (BI) prism flipper was used for near fusional vergence facility (VF).

Contact lens fitting included refractive correction, over refraction testing, confirmation of the central position and corneal coverage of the lenses. A slit lamp (Topcon DC3-slit lamp) with a digital video camera was used to assess whether the contact lens could completely cover the cornea and lens position when worn. Next, the tested lenses were used for contact lens fitting before refractive errors were performed to confirm the accuracy of the degree of the contact lens. The subjects were randomized into three groups and fitted with different contact lenses. Next, visual acuity and binocular vision were performed. Subjects were required to wear the contact lenses for more than 6 h a day, questionnaires of VBP and visual quality, and binocular vision examinations were conducted after 2 weeks and 4 weeks of wearing the lens.

The visual quality questionnaire contained 13 questions regarding lens comfort, visual clarity, diplopia or double vision, halos, and visual stability for assessing visual acuity fluctuation after wearing for a period of time. A higher score indicated better visual quality. Finally, an overall satisfaction score was assigned to the contact lens, which ranged from 0 to 10 points, where 0 was extremely dissatisfied and 10 was extremely satisfied.

### Data analysis and statistical analysis

The sample size of this study was determined by using G*Power analysis, under effect size d = 0.8, α = 0.05, power (1 − β) = 0.80. The calculation result of the total sample size was 21. Mixed design two-way analysis of variance (MANOVA) with main effects or simple main effects analysis (Bonferroni post hoc comparison) was performed using SPSS 26.0 statistical software (IBM Corp., Armonk, NY). A value of *p* < 0.05 was considered statistically significant.

### Institutional review board statement

The study was conducted according to the guidelines of the Declaration of Helsinki, and approved by the Ethics Committee of Chung Shan Medical University Hospital (Taichung, Taiwan) (Approval number: CS2-20089).

## Results

Nineteen subjects completed the study and were randomly divided into 3 groups. One-way analysis of variance (ANOVA) and nonparametric tests showed the absence of significant intergroup differences in age, VBP score, or refractive errors (Table 2). The number of participants that completed the study (19) was less than the sample size required (21); however, the power appeared to be adequate after recalculation and adjustment (effect size d = 0.5, α = 0.05, power (1 − β) = 0.952.).

**Table 2:** General information of study subjects.

Group	N	Age (year)	VBP (point)	Right Sph (D)	Right Cyl (D)	Left Sph (D)	Left Cyl (D)
Lens 1	6	21.67 ± 1.53	11.83 ± 3.31	− 3.79 ± 1.60	− 0.38 ± 0.38	− 3.46 ± 1.85	− 0.50 ± 0.35
Lens 2	7	21.49 ± 0.34	13.00 ± 3.51	− 2.61 ± 1.83	− 0.46 ± 0.22	− 2.14 ± 2.09	− 0.57 ± 0.37
Lens 3	6	21.62 ± 2.13	14.33 ± 5.20	− 2.67 ± 1.26	− 0.33 ± 0.20	− 2.71 ± 1.85	− 0.38 ± 0.34
*P* value		0.974	0.577	0.364	0.688	0.492	0.621

### Influence of contact lenses with different optical designs on binocular vision

#### Distance vergence

**Figure 2:**
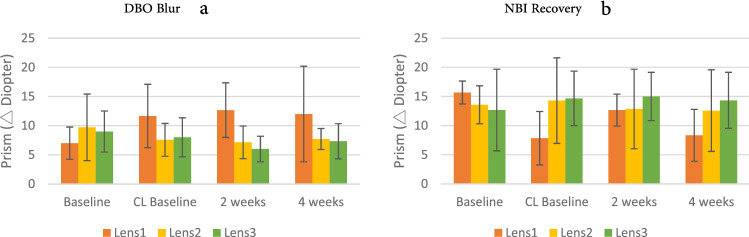
(**a**) DBO (Distance Base Out) blur; (**b**) NBI (Near Base in) recovery performance on Groups × Measurements; error bars represent the standard deviation.

Distance vergence examination included DBI break, DBI recovery, DBO blur, DBO break, and DBO recovery. Two-way ANOVA was used to analyze the four measurements and three lens groups of distance vergence. The results showed that the lens groups showed a significant interaction with 4 measurements of DBO blur (F = 3.346, *p* = 0.008). The BO prism was used to measure positive relative vergence, which is the ability of bilateral convergence to maintain a single clear image. Bonferroni post hoc analysis showed that the measurements on initially wearing the single-vision contact lenses (Lens 1 CL baseline) and after 2 and 4 weeks of wearing them were greater than the baseline value. In addition, the DBO blur value on 2 weeks and 4 weeks of wearing single-vision contact lenses (Lens 1) was also significant higher than that of wearing progressive (Lens 2) and bifocal (Lens 3) contact lenses, showing that different contact lens designs have a significant impact on the function of DBO blur. Wearing single-vision contact lenses may increase convergence demand, whereas wearing bifocal or progressive contact lenses might relax both eyes and reduce the convergence demand. (Fig. 2a).

#### Near vergence

Near vergence examination included NBI blur, NBI break, NBI recovery, NBO blur, NBO break, NBO recovery, NPC, and VF. Two-way ANOVA was used to analyze the four measurements and the three lens groups for near vergence. The results showed that the lens groups showed a significant interaction with 4 measurements on NBI recovery (F = 2.781, *p* = 0.021). The BI prism was used to measure negative fusional vergence, which is the ability of bilateral divergence to maintain a single visual image. Bonferroni post hoc analysis showed that the measurements on wearing single-vision contact lenses for the first time (CL baseline), 2 weeks, and 4 weeks were all less than the Lens 1 baseline value. Moreover, the NBI recovery value of single-vision contact lenses were significantly lower than that of progressive (Lens 2) and bifocal (Lens 3) contact lenses during CL baseline and 4 weeks of wearing (Fig. 2b), showing that different contact lens designs significantly impacted the function of NBI recovery. Wearing single-vision contact lenses might have an unstable performance, and also could affect fusional divergence in both eyes.

**Figure 3:**
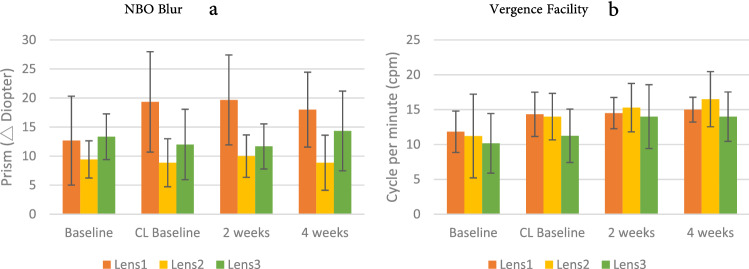
(**a**) NBO (Near Base Out) blur, (**b**) vergence facility performance on Groups × Measurements; error bars represent the standard deviation.

In addition, there were significant differences on NBI blur, NPC, and vergence facility between the four measurements; and also showed significant difference on NBO blur, NBO break, and NPC between the three lens groups. Although there was no significant interaction on NBO blur (F = 2.041, *p* = 0.078), Bonferroni post hoc analysis showed that the measurement when single-vision contact lenses (Lens 1) was initially placed, the NBO blur raised significantly higher than the Lens 1 baseline value (Fig. 3a), showing that wearing single-vision contact lenses could increase convergence demand, and stimulate binocular convergence. Although there was no significant interaction in vergence facility between the three contact lens groups, significant differences were present between the four measurements (F = 19.265, *p* = 0.000), indicating that not only single-vision contact lenses but also bifocal and progressive contact lenses (Fig. 3b) might be helpful for vergence facilities, especially progressive lenses (Lens 2).

#### Accommodation

**Figure 4:**
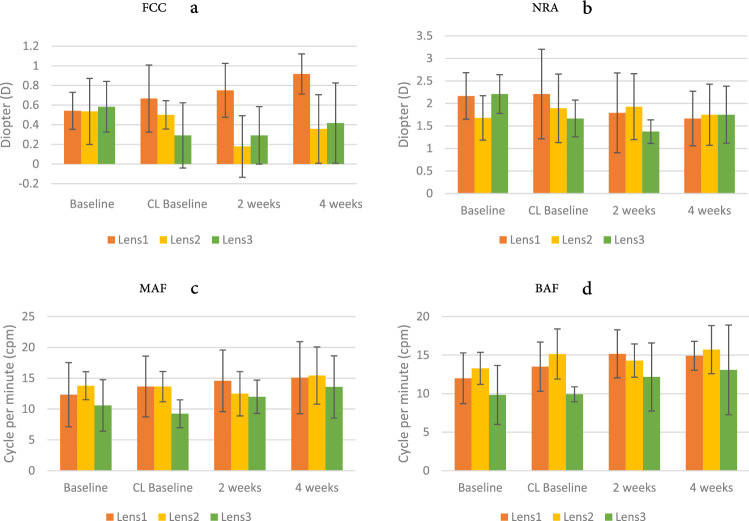
(**a**) Fused cross cylindrical (FCC) lens measurement; (**b**) negative relative accommodation(NRA); (**c**) MAF; (**d**) BAF performance on Groups × Measurements; error bars represent the standard deviation.

Accommodation examination included FCC, NRA, PRA, MAF, BAF, and AA. The results showed that FCC (F = 3.256, *p* = 0.009) and NRA (F = 3.539, *p* = 0.006) performed significant interactions on four measurements between different lenses. Bonferroni post hoc analysis indicated that the FCC significantly increased when the participants wore single-vision contact lenses (Lens 1). The FCC values of wearing progressive contact lenses (Lens 2) reduced significantly after 2 weeks and became much lower than that of the Lens 2 baseline and Lens 2 CL baseline (Fig. 4a). This was also observed in terms of the performance of Lens 3. This shows that accommodation lag might increase while wearing single-vision contact lenses; conversely, wearing progressive defocus contact lenses (Lens 2) and bifocal contact lenses (Lens 3) could reduce lag of accommodation and theoretically the positive relative accommodation or fusional vergence at near.

Regarding NRA (Fig. 4b), the measurement at 4 weeks of wearing single-vision contact lens (Lens 1) was significantly lower than the Lens 1 baseline value. In the bifocal contact lens (Lens 3) group, the NRA measurements were also significantly reduced from the lens 3 CL baseline and 2 weeks of wearing, Lens 2 NRA was maintained in a relatively stable state. NRA tests positive relative vergence (PRV), and this result shows that wearing single-vision (Lens 1) and bifocal (Lens 3) contact lenses could compromise the convergence (PRV). Despite the absence of a significant interaction between in BAF and MAF (binocular and monocular accommodative facility) in the lens groups, there were significant differences in BAF and MAF accommodative facility in the four measurements (MAF: F = 3.397, *p* = 0.025, Fig. 4c; BAF: F = 6.949, *p* = 0.001, Fig. 4d), showing trends of improvement, especially in the Lens 3 group.

#### Fixation disparity, and horizontal phoria

**Figure 5:**
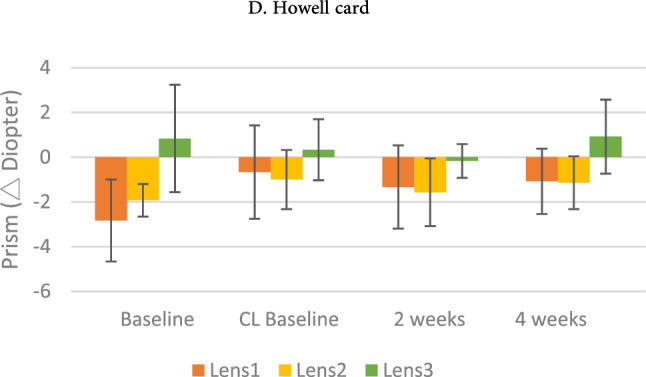
Distance Howell card performance on Measurements × Groups; error bars represent the standard deviation.

Two-way ANOVA was performed on the four measurements and three groups for fixation disparity and horizontal phoria. Both distance and near fixation disparity and horizontal phoria showed significant differences in the three contact lens groups. Only distance phoria showed significant differences before and after wearing contact lenses, Howell card performance at 3 m (F = 2.507, *p* = 0.034) showed significant interactions on Groups x Measurement. Simple main effects analysis revealed that all three contact lenses showed a tendency to alter the condition of the phoria. Comparing the distant phoria amount at baseline with that after wearing the contact lenses for 2–4 weeks revealed a trend was considered a significant Orthophoric development in both single-vision contact lenses (Lens 1) and progressive contact lenses (Lens 2); however, this trend was not observed in bifocal contact lenses (Lens 3). (Fig. 5).

#### Modulation transfers function (MTF)

**Figure 6:**
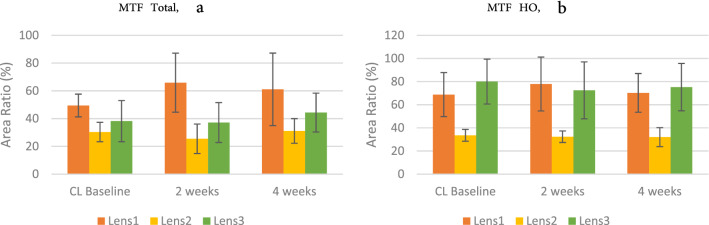
(**a**) Total and (**b**) higher order aberration area ratio in MTF performance on Measurements × Groups; error bars represent the standard deviation.

The MTF can be used to predict the contrast sensitivity, since the MTF is simulated from aberrations measured, Optical Path Difference Scanning System (OPD3, Nidek) was performed in the full-dark room to obtain aberration variances, data of 5 mm pupil were collected for analysis. Since the MTF measurement cannot be performed when wearing frame glasses, only CL baseline, 2 week, and 4 week data were compared. Two-way ANOVA showed that there was no significant interaction on the total area ratio (F = 1.735, *p* = 0.193, Fig. 6a) and higher-order aberration area ratio (F = 0.105, *p* = 0.901, Fig. 6b), but the MTF showed significant difference between three lenses (F = 3.042, *p* = 0.028). A progressive defocus contact lens (Lens 2) showed that a progressive defocus design may interfere with the total MTF (Lens 1 vs. Lens 2: *p* = 0.000; Lens 2 vs. Lens 3: *p* = 0.511), and HO MTF (Lens 1 vs. Lens 2: *p* = 0.000; Lens 2 vs. Lens 3: *p* = 0.000), resulting in decreased contrast sensitivity. However, whether MTF can accurately predict subjective contrast sensitivity requires elucidation.

### Influence of contact lenses with different optical designs on visual behavior performance

To analyze the effects of contact lenses with different optical designs on visual behavior performance before and after wearing contact lenses, participants were asked to complete the questionnaire at the beginning of the experiment and the final measurement (4 weeks). A negative value implied that the visual behavior improved (post–pre), and the more negative the score was, the greater the improvement. Next, differences in the total visual behavior score and four dimensions before and after wearing the three contact lenses were analyzed. The results showed that there were no statistically significant differences in the total VBP score or various dimensions between the three contact lenses.

**Figure 7:**
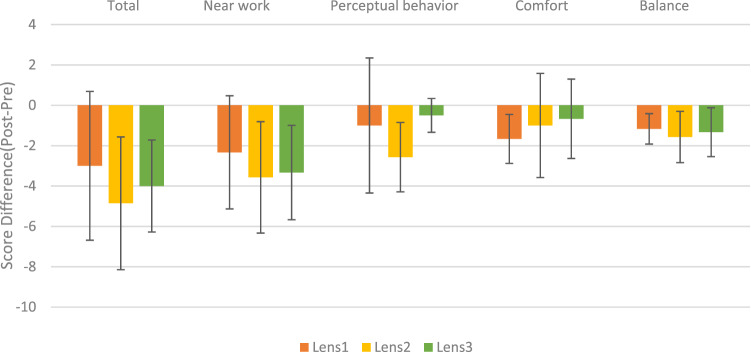
Visual behavior performance score before and after wearing contact lenses; error bars represent the standard deviation.

Although one-way ANOVA analysis showed that no significant differences were found in all items (Total: F = 0.561, *p* = 0.581; Near work: F = 0.385, *p* = 0.687; Perceptual: F = 1.597, *p* = 0.233; Comfort: F = 0.375, *p* = 0.693; and Balance: F = 0.217, *p* = 0.807), the mean values were all negative, showing that visual behavior showed varying degrees of improvement after wearing the three contact lenses. The extent of improvement was compared (Fig. 7), and the results showed that the progressive defocus contact lens (Lens 2) was the most beneficial lens for overall visual behavior and showed the best results for near work, perceptual behavior, and posture balance. This was followed by the bifocal contact lens (Lens 3), which was beneficial for near work and posture balance in addition to overall performance. Single-vision contact lenses (Lens 1) showed better performance in comfort than the other two contact lenses.

### Influence of contact lenses with different optical designs on visual quality

After wearing contact lenses for 4 weeks, the visual quality questionnaire was completed during the last follow-up measurement to assess the effects of the three contact lenses on visual quality (Table 3). One-way ANOVA statistical results showed that there were significant differences in terms of nocturnal halos (F = 13.562, *p* = 0.000); Bonferroni post hoc: Lens 1 > Lens 2 (*p* = 0.002), Lens 3 > Lens 2 (*p* = 0.000). This shows that progressive defocus contact lenses may have poorer nocturnal halos at night.

**Table 3:** Effects of the three contact lenses on visual quality.

		Group	Mean	SD	N	F	*p*	Post hoc
Diplopia or Double vision	Distance	Lens 1	4.50	0.837	6	1.436	0.267	−
		Lens 2	4.14	0.690	7			
		Lens 3	3.67	1.033	6			
	Near	Lens 1	4.33	0.816	6	0.380	0.690	−
		Lens 2	4.29	0.756	7			
		Lens 3	3.83	1.602	6			
Visual Clarity	Distance	Lens 1	4.33	0.816	6	1.838	0.191	−
		Lens 2	3.57	0.787	7			
		Lens 3	3.33	1.211	6			
	Middle	Lens 1	4.50	0.837	6	0.353	0.708	−
		Lens 2	4.14	0.690	7			
		Lens 3	4.17	0.983	6			
	Near	Lens 1	4.50	0.837	6	0.574	0.574	−
		Lens 2	4.14	0.690	7			
		Lens 3	4.50	0.548	6			
Halo	Day	Lens 1	4.00	1.265	6	0.730	0.497	−
		Lens 2	4.43	0.787	7			
		Lens 3	4.67	0.816	6			
	Night	Lens 1	3.83	0.753	6	13.562	0.000*	Lens 1 > Lens 2 (*p* = 0.002)
		Lens 2	2.43	0.976	7			
		Lens 3	4.67	0.516	6			Lens 3 > Lens 2 (*p* = 0.000)
Visual stability	Distance	Lens 1	4.50	0.548	6	3.436	0.051	−
		Lens 2	3.57	0.535	7			
		Lens 3	3.83	0.753	6			
	Middle	Lens 1	4.83	0.408	6	2.394	0.123	−
		Lens 2	4.00	0.816	7			
		Lens 3	3.83	1.169	6			
	Near	Lens 1	4.67	0.516	6	1.990	0.169	−
		Lens 2	4.00	0.577	7			
		Lens 3	4.00	0.894	6			
Satisfaction		Lens 1	8.17	0.753	6	1.833	0.192	−
		Lens 2	7.43	0.535	7			
		Lens 3	6.83	1.941	6			

## Discussion

Compared with spectacles, single-vision contact lenses may increase convergence and accommodative demand in both eyes, causing more accommodation and excessive convergence focusing on near objects^20^. According to this study, wearing single-vision contact lenses (Lens 1) might induce a larger lag of accommodation, affect NRA, stimulate more accommodation for convergence and esophoria, and decrease negative fusional reserve near^16^, which is consistent with the viewpoints in the literature. However, single-vision contact lenses did not show significant changes in distant vergence, vergence facilities, or accommodative facilities.

Wearing the progressive (Lens 2) and bifocal (Lens 3) contact lenses had similar variation trends, such as reduced lag of accommodation and contrast sensitivity^29,39–42^. A progressive lens affects near vergence and improves vergence and accommodative facilities. A progressive defocus design lens may improve peripheral refractive status and decrease accommodative lag^14,15^. In addition, related studies on multifocal contact lenses found the effects of exophoria shifting and deterioration of stereo vision^24^; however, halos in the night was one of the problems of progressive lenses. Although previous studies have reported changes in contrast sensitivity with multifocal contact lenses of similar designs^27,28^, whether the subjective MTF values can predict subjective contrast sensitivity remains to be discussed.

Comparison of the three lenses before and after wearing demonstrated improvements in vergence and accommodative facility. Vesely et al. found that progressive contact lenses and low addition of + 0.50 D could improve accommodative and vergence facilities in subjects with eye fatigue^43^. However, previous studies on single-vision contact lenses did not find any significant differences in accommodative facilities. Single-vision contact lenses affect binocular fusional divergence and increase accommodative lag^23^. A paper has shown that peripheral refraction is related to accommodation^43,44^, and wearing progressive contact lenses may help relax accommodation and decrease accommodative lag^24^. However, the peripheral defocus optical design may interfere with peripheral vision, which might decrease contrast sensitivity^29,40^. On the other hand, wearing single-vision contact lenses increases accommodative demand^20^. Both bifocal and progressive defocus contact lenses showed convergence relaxation trends, and changes in eye position only occurred in esophoria in the single-vision contact lens group^23^. Furthermore, single-vision and bifocal contact lenses resulted in reduced NRA. NRA/PRA are important markers that affect accommodation and vergence in both eyes and are used to measure the ability of both eyes to maintain a single-vision^45^. Felipe-Marquez et al. assessed the effects of orthokeratology lenses on accommodation and found that NRA significantly increased after long-term (3-year) follow-up^46^, showing significant improvement in accommodation^21^. This shows that contact lenses with different optical designs affect NRA, but the variation trends and mechanism still require further study.

Analysis of the subjective feelings of the subjects found that single-vision contact lenses have better performance and fewer effects on visual behavior^47^, whereas subjects who wore progressive and bifocal contact lenses mentioned that there was significant improvement in overall visual behavior. However, most papers showed a decrease in visual performance, a slight decrease in visual acuity, poorer visual quality and comfort, and decreased reading capacity and speed^47–50^. The reasons for these differences may be attributable to subject selection. In this study, subjects with poor visual behavior were recruited, which was different from the enrollment criteria of other studies. This study might be a good start to discuss whether multifocal or bifocal contact lenses are more suitable for subjects with abnormal binocular vision.

Next, we reviewed the visual quality of the three contact lenses: single-vision contact lenses have the best lens comfort^48^; progressive defocus lenses tend to cause nocturnal halos^51^ ; and bifocal contact lenses simultaneously converge light rays from two optical zones to achieve synchronous vision. When the main object is clear and in focus, the image in the other optical zone becomes a blurred background^52^. This presents as diplopia or double vision. In addition, the distance vision clarity and stability of progressive defocus and bifocal contact lenses may be affected by the peripheral design of the lenses and are slightly decreased^48,50^. Based on the actual scores assigned by subjects, single-vision contact lenses have the best overall satisfaction, followed by progressive defocus contact lenses and then bifocal contact lenses.

## Conclusion and limitations

Compared with single-vision contact lenses, progressive and bifocal contact lenses may be improve binocular vision function, and visual behavior performance, but there may be adaptation problems in the wearing process. Prescription shall be made according to the purpose of personal wear and binocular vision functions. The limitations of this study are: (1) Small sample size: Even though the study showed significant changes, there was insufficient statistical power to generalize the results to the whole population; (2) Difficulty in subject recruitment: as the inclusion criterion was subjects with poor visual behavior, a large number of questionnaires was required for initial screening to identify subjects who met this criterion; (3) Identities of subjects: The subjects recruited in this study were university students and their daily eye usage requirement might have differed, e.g., near work duration may drastically increase when report submission deadlines are approaching or before examinations, which might have affected binocular vision measurements; (4) Study duration: This one-and-a-half-month long study and four follow-up measurements might have decreased the participation willingness of many subjects. However, the 4-week wearing period might be insufficient to support changes in long-term wearing, and a longer follow-up study may be required.

We recommend that subjects with normal binocular vision should be first enrolled in subsequent studies to increase the sample size and analyze the effects of contact lenses with different optical designs on binocular vision. Following this, items with significant effects or specific populations may be further analyzed. In addition, lenses with different near ADDs could be compared to examine the effects of addition on binocular vision and visual behavior.

## Supplementary Information

Supplementary Information.

## Data Availability

The datasets used during the current study are available from the corresponding author.
