# Supplementary material for: The influence of contact lenses with different optical designs on the binocular vision and visual behavior of young adults
Source: Sci Rep. 2022 Apr 21;12:6573. doi: 10.1038/s41598-022-10580-7 (PMC9023515; doi:10.1038/s41598-022-10580-7)
Supplement: Supplementary file 1 — Supplementary Information. [file 41598_2022_10580_MOESM1_ESM.pdf]

Appendix 1

CSMU - Visual Behavioral Performance<br>If you have the following situation, please put a “v” in the “underline”	
1. ___Skipping/repeating lines while reading	25. ___Feel difficult to pursuit
2. ___Losing your place when reading or writing	26. ___ Uncoordinated extremity
3. ___Omitting words when reading or writing	27. ___Uncoordinated movement
4. ___Making mistake when reading or writing	28. ___Eyes turn to outward or inward
5. ___Have to repeat reading	29. ___One eye is clearer than the other
6. ___Upside-down numbers	30. ___Feel sleepy when reading
7. ___Using fingers to follow words when reading	31. ___Poor sense of direction
8. ___Slow reading	32. ___Slow copying
9. ___Poor reading comprehension	33. ___Poor visual motor integration
10. ___Difficulty remembering what you have read	34. ___Easier to speak than write
11. ___Holding books or near work very close to eye	35. ___Avoiding task with more attention
12. ___Closing one eye when reading	36. ___Avoiding near work or reading
13. ___Tilting head when reading	37. ___Struggle to tell tight from left
14. ___Headache with reading or using computer for a while	38. ___Getting anxious when working on others desk
15. ___ Eye pain with reading or using computer for a while	39. ___Ignore surrounding when concentrate on something
16. ___ Extreme fatigue with reading or using computer for a while	40. ___Wired handwriting posture
17. ___Double vision when reading or near work	41. ___Abrasion of shoes at outward or inward
18. ___Blur at far while near work for a period	42. ___Motion sickness
19. ___Words run together while reading	43. ___Photophobia
20. ___Short attention span with near work	44. ___Excessive eye blinking
21. ___Poor spelling	45. ___Rubbing your eyes often
22. ___Writing uphill or downhill	46. ___Dry eyes
23. ___Misaligning digits/columns of numbers	47. ___Watery eyes
24. ___Difficulty copying from a chalkboard	48. ___Eye redness
